# Commentary on 'What is the point: will screening mammography save my life?' by Keen and Keen

**DOI:** 10.1186/1472-6947-9-19

**Published:** 2009-04-02

**Authors:** Stephen W Duffy

**Affiliations:** 1 Cancer Research UK Centre for EMS, Wolfson Institute of Preventive Medicine, Barts and the London School of Medicine and Dentistry, Charterhouse Square, London, EC1M 6BQ, UK

## Abstract

Commentary on Keen and Keen 'What is the point: will screening mammography save my life?' BMC Medical Informatics and Decision Making, 2009

## Commentary

The paper by Keen and Keen [1] presents new estimates of the absolute numbers of breast cancer deaths prevented by mammographic screening. The estimation is carried out by synthesis of a range of estimates of relative risk of breast cancer mortality loosely derived from the randomised trials, SEER data on breast cancer mortality and the proportion of the recent reduction in breast cancer mortality estimated by Berry et al to be owing to mammography [2]. Much is made of the relatively small absolute benefit estimated, and indeed the absolute benefit estimated here is notably smaller than that estimated in a randomised trial, and in an evaluation of service screening [3,4]. In relation to the results and the accompanying discussion, two remarks spring to mind.

The first is that the paper rather labours the obvious point that in breast cancer screening, as in primary and secondary prevention generally, one has to apply the intervention to large numbers of healthy subjects in order to benefit the few who are unlucky enough to develop the disease. The same argument can be made of vaccination, cervical screening and many other interventions. If one is in the business of preventive medicine, one has to accept this as a fact of life. The improvements in length of life in recent decades are a combination of two per thousand from one disease, three per thousand from another, and so on.

The second point is that the accuracy of the figures arrived at is questionable. From a rather convoluted ecological synthesis of information from different sources, the authors arrive at the finding that 1.8 breast cancer deaths would be prevented by repeatedly screening 1000 women for 15 years. Directly estimating this from empirical randomised trial data gives a figure of approximately 3 per 1000 over ten years [3], and from service screening in Sweden 2.1 per 1,000 [4]. They also estimate that less than 5% of screen-detected cases have their lives saved as a result of the screening. This is clearly at odds with the experimental evidence. In the Swedish Two-County Trial, 141 breast cancer deaths were prevented, 15% of the 928 screen-detected cancers.

Why are the authors' modelled results different from the empirically observed results of others? One reason may be the difference between the time frames of the authors' model and the period of observation in the empirical data. Another may be their underestimation of the relative benefit of screening. The randomised trials combined yield an estimate of a 20% mortality reduction with ***invitation ***to screening [5], whereas the authors posit this as the reduction conferred by actually ***receiving ***screening. A more subtle reason may be the authors' procedure of first removing the deaths from breast cancer estimated to be prevented by innovations in therapy, then applying the relative reduction from screening to the remaining deaths. The latter was estimated using all tumours in the trials, not just for whom death had not been prevented by specific treatments. The relative mortality reduction from mammography in the subgroup of cases whose lives are not saved as a result of treatment is likely to be considerably larger than the overall estimated reduction.

In any case, whatever the reason for the differences, when there is a disagreement between direct results from empirical data and modelled estimates derived by combining information from disparate sources, it would be wise to trust the former.

## Competing interests

The author declares that he has no competing interests.

## Pre-publication history

The pre-publication history for this paper can be accessed here:

http://www.biomedcentral.com/1472-6947/9/19/prepub
